# Error-correcting properties of the SOLiD Exact Call Chemistry

**DOI:** 10.1186/1471-2105-13-145

**Published:** 2012-06-22

**Authors:** Tim Massingham, Nick Goldman

**Affiliations:** 1 European Bioinformatics Institute, Wellcome Trust Genome Campus, Hinxton, Cambridgeshire, UK

## Abstract

**Background:**

The Exact Call Chemistry for the SOLiD Next-Generation Sequencing platform augments the two-base-encoding chemistry with an additional round of ligation, using an alternative set of probes, that allows some mistakes made when reading the first set of probes to be corrected. Additionally, the Exact Call Chemistry allows reads produced by the platform to be decoded directly into nucleotide sequence rather than its two-base ‘color’ encoding.

**Results:**

We apply the theory of linear codes to analyse the new chemistry, showing the types of sequencing mistakes it can correct and identifying those where the presence of an error can only be detected. For isolated mistakes that cannot be unambiguously corrected, we show that the type of substitution can be determined, and its location can be narrowed down to two or three positions, leading to a significant reduction in the the number of plausible alternative reads.

**Conclusions:**

The Exact Call Chemistry increases the accuracy of the SOLiD platform, enabling many potential miscalls to be prevented. However, single miscalls in the color sequence can produce complex but localised patterns of error in the decoded nucleotide sequence. Analysis of similar codes shows that some exist that, if implemented in alternative chemistries, should have superior performance.

## Background

The collection of technologies described as Second- or Next- Generation Sequencing (NGS) platforms are characterised by the synthesis of complementary strands of DNA from clusters of homologous templates [1]. The chemistry used differs between the platforms but that for the Life Technologies Corporation SOLiD platform is particularly interesting since there is not a one-to-one correspondence between measurements made during sequencing and nucleotides of the sequence being read. Instead the primary output of the SOLiD platform is a ‘color sequence’, an encoded form of the nucleotide sequence, that has advantages for calling SNPs when comparing the reads to a reference [2]. With the advent of the 5500 Series of machines in November 2010, an improved ‘Exact Call Chemistry’ (ECC) was introduced that changes the way that the sequence is encoded by the platform and allows mistakes in the measurements to be corrected, hence producing more accurate reads [3].

The SOLiD sequencing chemistry consists of multiple rounds where probes, consisting of eight bases and a fluorophore, are sequentially ligated to the template sequence to build up a complementary strand. Each round consists of a priming step followed by a repeated cycle of ligating probes to the template, exciting the fluorophores and imaging the resulting emission, then cleaving the flurophore and part of the probe ready for the next cycle. The probe/fluorophore combinations are designed so that the probes interrogate the first two of the eight ligated positions in the template, with each of four fluorophore colors used to indicate four of the 16 possible nucleotide pairs at these positions. The color of the fluorophore for each template is recorded and used later to determine the sequence of the read. After imaging, those templates to which a probe failed to ligate have their previous probe decapped (i.e. dephosphorylated) so they will not be extended on future cycles. This reduces problems analogous to ‘phasing’ on the Illumina platform [4]. The fluorophore and last three bases are cleaved from the probe, leaving the strand ready to be further extended in the next cycle. The repeated ligation of eight additional bases and then cleaving the end three mean that the pair of bases of the template sequence that is interrogated moves on by five positions every cycle. After a specified number of cycles, the round is stopped and the complementary strand melted from the template to leave the template ready to be primed for the next round. Each round uses a different primer so that the positions interrogated by the probes change each time; for example, on the first round the first position of the probes on cycles 1, 2, … corresponds to positions 1, 6, … of the template sequence; on round two these become positions 2, 7, …, etc.

After five rounds, probes have been ligated so that every position of the template sequence has been the first position of a probe. The colors recorded and prior knowledge of one of the bases, typically the last adapter base adjacent to the template, are sufficient to determine the nucleotide sequence assuming that no errors have been made. Conventionally, one of the rounds is primed so that it starts one base before the beginning of the template sequence and so the first base interrogated is the last base of a known adapter sequence.

The SOLiD Exact Call Chemistry (ECC) augments the ‘two-base-encoding’ chemistry with an extra round where a different set of probes is ligated. Each cycle of this round interrogates positions 1, 2 and 4 of each five-nucleotide block of the template. The same four fluorophores are used, each now indicating the presence of one of 16 of the possible 64 combinations of nucleotides at the positions interrogated. These color calls can be used to detect and recover from miscalls made in previous rounds.

Directly decoding the colors from the first five rounds into nucleotide sequence allows catastrophic failures to occur where a single calling error creates errors in every position from then on [2]. As well as allowing some errors to be corrected, the additional round also enables the calls to be translated into nucleotide sequence in a manner that behaves more gracefully in the presence of mistakes [3].

The mathematical basis for the error correcting properties of the SOLiD ECC is the theory of convolutional codes [3], a class of codes which are incorporated into some of the most powerful error-correcting codes in general use, for example those used in the Voyager deep-space exploration program [5]. Convolutional codes are a category of linear codes [6,7] and we use the theory of linear codes to analyse the error correcting properties of the SOLiD Exact Call Chemistry, describing the types of sequencing mistakes it can correct and those cases where the presence of an error can only be detected. For isolated mistakes that cannot be unambiguously corrected, we show that the type of substitution can be determined and its location can be narrowed down to two or three positions in the read, leading to a significant reduction in the the number of plausible alternative reads. If the read is ‘corrected’ at the wrong location, we show that the nucleotide read ultimately produced contains errors that have a distinctive pattern. Finally, we apply the same techniques used to analyse the Exact Call Chemistry to look at hypothetical alternative chemistries and show that some of them have superior characteristics, being able to correct more errors and inducing simpler patterns of error in the decoded nucleotide sequence when reads are miscorrected.

## Methods

### Convolutional code theory

We start by defining the terminology we need to analyze the SOLiD code. The chemistry of the sequencing reactions and imaging mean that each fragment of DNA, or ‘source sequence’ (nucleotides), is encoded into a ‘code sequence’ (colors) of equal or greater length. The original five rounds produce as many observed colors as there are nucleotides in the fragment; the ECC round adds one-fifth as many additional color calls. The code sequence is then observed with potential errors (‘observed sequence’). Since the code sequence is longer than the source sequence, not all possible code sequences correspond to an encoded source sequence; those that do correspond are termed ‘valid’. Given an observed sequence, some procedure is used to find the closest valid code sequence (‘corrected sequence’) and the nucleotide sequence that produces this code sequence is the decoded sequence. Note that the source sequence and decoded sequence are sequences of nucleotides (‘base-space’), whereas the code, observed and corrected sequences are colors (‘color-space’).

In terms of the theory to be presented, colors and nucleotides are just different representations of the same things, members of a set of four elements over which a finite field (i.e. Galois field, GF_4_) is defined (see Table 1). To prevent confusion between the various matrices and vectors studied and their concrete representation as nucleotides or colors, all calculations in the text are described in terms of elements GF_4_ and the more usual notations are reserved for when an actual sequence of nucleotides or colors is meant.

**Table 1:** Different representations of GF_4_

**Representation**	**Elements**	*⊕*	**0**	**1**	*α*	*β*	*⊗*	**0**	**1**	*α*	*β*			
GF_4_	0	1	*α*	*β*	0	0	1	*α*	*β*	0	0	0	0	0
Nucleotides	A	C	G	T	1	1	0	*β*	*α*	1	0	1	*α*	*β*
Colors	***0***	***1***	***2***	***3***	*α*	*α*	*β*	0	1	*α*	0	*α*	*β*	1
					*β*	*β*	*α*	1	0	*β*	0	*β*	1	*α*

For the purposes of the coding theory presented here, both nucleotides and colors represent elements of the Galois Field over four elements (GF_4_) and the correspondence between them is shown below. For example, the color ‘***2***’, the nucleotide ‘G’ and the element ‘*α*’ are considered to be equivalent for the purposes of calculation. A field consists of a set of elements and rules on how to add (⊕) and multiply (⊗) them together; the results of combining two elements are expressed by the Cayley tables above; for example, *α*⊕*β*=1 and *α*⊗*α*=*β*. The standard rules for associativity and commutativity for multiplication and addition still apply in finite fields, and multiplication is still distributive over addition [8]. One notable difference from ordinary arithmetic is that all elements are self-invertible under addition in GF_4_, so addition and subtraction are equivalent operations.

The ECC code, and any linear code in general, is defined in terms of a *k*×*n*generator matrix *G* with elements in GF_4_. A source sequence *s* is a row vector of length *k* consisting of elements of GF_4_, and its code sequence *c* is *sG* where the vector-matrix multiplication is understood in the usual sense but addition and multiplication of elements is carried out within GF_4_ (Table 1). Codes are termed linear because any linear combination of valid code sequences is also a valid code sequence: if _*s*1_ and _*s*2_ are source sequences with corresponding code sequences _*c*1_and _*c*2_, and _*w*1_ and _*w*2_ are elements of GF_4_ then _*w*1__*c*1_ + _*w*2__*c*2_=(_*w*1__*s*1_ + _*w*2__*s*2_)*G* so *c*=_*w*1__*c*1_ + _*w*2__*c*2_ is a valid code sequence corresponding to the source sequence _*w*1__*s*1_ + _*w*2__*s*2_.

Rather than expressing errors in terms of a specific color miscall, we consider types of errors defined by what happens when an element of GF_4_ is added to a call. For example, the error type ‘ + *β*’ transforms the color ***1*** (≡1, an element of_GF4_) into ***2*** (≡*α*) since 1⊕*β*=*α*, and transforms ***0*** (≡0) into ***3*** (≡*β*) since 0⊕*β*=*β*(see Table 1). Although we will be considering color miscalls in the coding sequence, it is instructive to examine the action of these error types on nucleotides to show that they can have a concrete interpretation: + *β* complements the nucleotide, + *α*results in a transition, +1 preserves whether the nucleotide is an amino- or keto- acid (‘transcomplement’) and +0 leaves the nucleotide unchanged. The group structure ensures that all possible substitutions can be encoded in this form and, when errors are rare, the sequence of error types will consist mostly of 0s. Adding the sequence of error types to the observed sequence results in the recovery of the (true) code sequence; likewise, adding the error types to the code sequence results in the observed sequence.

Each generator matrix has an associated parity check matrix *H* that can be used to determine whether a code sequence is valid. The parity check matrix uses a subset of the elements of observed sequence to call a putative decoded sequence and then calculates the expected value of the remaining ‘parity’ elements by treating the decoded sequence as true. The expected and observed parity sequences are then summed element-wise to create the parity check sequence. *H* is constructed so that the parity check sequence is the sequence-matrix product *xH*, where *x* is the observed sequence. By construction, the parity check of valid code sequence is a sequence of 0s; invalid code sequences have a non-zero parity check. When errors have occurred, the observed sequence can be expressed in terms of the code sequence for the source sequence and a sequence of error types *e*: *x*=*c* + *e*. Since the parity check of a valid code sequence is a sequence of zeros, the parity check of the observed sequence only depends on the sequence of errors and not the source sequence as *xH*=(*c* + *e*)*H*=*eH*. The value of *eH*=*xH* is termed the ‘syndrome’ of the error, a sequence in GF_4_ of length *n*−*k*that partitions the set of possible errors into equivalence classes.

Syndromes provide a simple and efficient method of decoding an observed sequence under the assumption that errors are rare and so the most plausible corrected sequence is the valid coding sequence which requires the fewest changes from the observed sequence. An error consisting of multiple changes may belong to the same equivalence class as one with fewer changes but the simpler error is the more likely and so is the best predictor of what error actually occurred. Before decoding begins, a table is constructed mapping every syndrome (*σ*) to the simplest possible sequence of error types that has it (_*e* *σ*_); this can be done by enumerating all single errors, followed by doublets, triplets, etc. until every syndrome has been observed at least once and all error types up to a given complexity have been considered. The syndrome table is a function of the code structure only and can be calculated once and distributed with the probe sets. To decode each observed sequence *x*, its syndrome *xH* is calculated and then located in the table to find the simplest sequence of error types _*e* *xH*_ that could cause it. The sequence *x* + _*e* *xH*_is then a valid code sequence which can then be decoded. If there are several equally simple sequences of error types, then an error has been detected but the correction is ambiguous.

**Figure 1:**
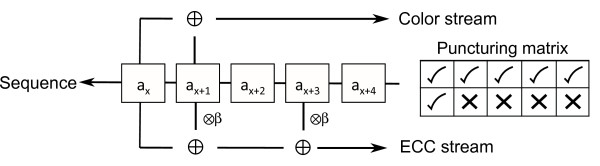
**Architecture of the SOLiD ECC encoder.** The architecture of the convolutional code for the SOLiD ECC consists of two streams. The nucleotide sequence _*a*1_,_*a*2_,… is passed through the encoder, progressing one position at a time, and the color obtained from the additions and multiplications indicated is emitted from each stream. The top ‘color stream’ is that produced by the two-base-encoding chemistry and the bottom ‘SOLiD ECC stream’ is punctured so that only every fifth color member of the sequence is used.

The ECC code is defined by the architecture shown in figure 1. The encoding ‘machinery’ looks at windows of length five of the source sequence, moving along one element at a time, and performing the specified additions and multiplications. Two streams of symbols are emitted. For the first stream, corresponding to the standard color encoding, the first two elements of the window are added together. For the second stream, the ECC encoding, only every fifth symbol is recorded, a practice known as puncturing or perforating. This stream multiplies the second and fourth elements of the window by *β* and sums them with the first element. In terms of the SOLiD chemistry rounds, the color calls from each of the first five rounds correspond to every fifth element of the first stream with a different offset for each round and the calls from the final round correspond to the punctured second stream.

The calculation for both the first and second streams (*i*=1,2) can be expressed as the dot product *a*·_*ρ* *i*_ where *a* is a row vector of the bases in the window and the _*ρ* *i*_ describe the calculations to be done, with _*ρ*1_=11000 and _*ρ*2_=1*β*0*β*0. The _*ρ* *i*_are the ‘probe generators’ for the chemistry since they define the color of fluorophore that each probe has attached to it: the probe that interrogates the sequence *b* has color *b*·_*ρ* *i*_.

**Figure 2:**
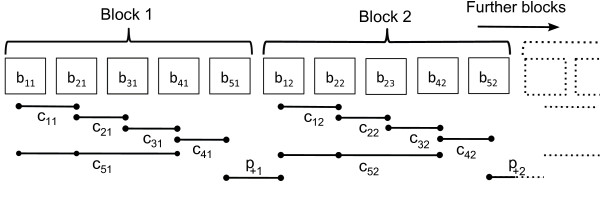
**Block structure of encoded read.** A read partitioned into blocks of five bases, with block *i* containing bases _*b*1*i*__*b*2*i*__*b*3*i*__*b*4*i*__*b*5*i*_, showing how the two-base-encoding and ECC color calls are split into five ‘data’ colors _*c*1*i*__*c*2*i*__*c*3*i*__*c*4*i*__*c*5*i*_, from which the block can be called, and a ‘parity’ color (_*p* + *i*_) which straddles the block and its downstream neighbour. The data colors are used to determine the nucleotide sequence of the blocks and the parity color is used to detect whether an error has occurred. Note that the data colors are a mixture of both color streams, with the parity color coming from the color stream of the code.

The architecture of the ECC code imposes the block structure shown in figure 2 on the source sequence. The read is partitioned into blocks of length five, each of which can be uniquely recovered from the indicated five elements of the encoded sequence. The remaining elements of the encoded sequence each span two blocks and are in effect parity colors that can be used to detect the presence of errors. Ignoring the parity colors, the generator for each block is the invertible matrix

(1) $$G_{\text{Block}}=\left(\begin{array}{c} 1\;0\;0\;0\;1\\ 1\;1\;0\;0\beta \\ 0\;1\;1\;0\;0\\ 0\;0\;1\;1\\ 0\;0\;0\;1\;0 \end{array}\right)\;G_{\text{Block}}^{-1}=\left(\begin{array}{c} 0\;1\;1\;1\;1\\ \beta \;\beta \;\alpha \;\alpha \;\alpha \\ \beta \;\beta \;\beta \;\alpha \;\alpha \\ 0\;0\;0\;0\;1\\ 1\;1\;1\;1\;1 \end{array}\right)$$

which can be used to freely convert between a sequence of five nucleotides and its encoding. For example, the nucleotide sequence ACGAT (≡01*α*0*β*) has the color encoding ***13233*** (≡1*βαββ*) since (01*α*0*β*)_*G*Block_=(1*βαββ*). Conversely, $\left(1\beta \alpha \beta \beta )G_{\text{Block}}^{-1}=(01\alpha 0\beta \right)$.

After reordering the encoded sequence appropriately, the generator for the full code, _*G*ECC_, can be expressed in terms of the generators for each of the blocks and an additional column for each of the parity colors:

(2) $$\;G_{\text{ECC}}=\left(\left(\begin{array}{c} G_{\text{Block}}\;0\;\ldots \\ \;\;0\;\;G_{\text{Block}}\;0\;\ldots \\ \;\;\vdots \;0\;G_{\text{Block}}\;\ddots \\ \;\vdots \;\ddots \;\ddots \end{array}\right)\right)\left(\left|\begin{array}{c} u_{5}\;0\;\;\ldots \\ u_{1}\;u_{5}\;\ddots \\ 0\;u_{1}\;\ddots \\ \vdots \;\ddots \;\ddots \end{array}\right)\right)=\left(G^{*}|P\right)$$

where _*u* *i*_is a column vector with five elements consisting of 0s except for a 1 in the ^*i*th^position, with G^*^ and *P* being defined appropriately. The dimensions of _*G*ECC_ are *k*×*n*, where *k* is the length of the read (source sequence) and *n* the total number of color calls produced in both the two-base-encoding and ECC chemistry rounds.

The parity-check matrix, _*H*ECC_, corresponding to generator _*G*ECC_, is

(3) $$H_{\mathrm{ECC}}=\left(\begin{array}{c} G^{*-1}\;\;0\\ \;0\;I_{n-k} \end{array}\right)\left(\begin{array}{c} P\\ I_{n-k} \end{array}\right)$$

where _*I* *m*_ is the *m*×*m*identity matrix. By considering the action of this parity-check matrix on an encoded sequence, the calculation of the syndrome can be given concrete form: the first matrix of the factorised form of _*H*ECC_ takes the observed sequence and inverts it block by block to get a putative nucleotide decoding, with the values of the parity colors preserved. The second matrix calculates the parity colors for the putative decoding and adds them element-wise to the parity colors actually observed. The resulting sequence of length *n*−*k* is the syndrome. If the syndrome is composed entirely of 0s, i.e. the parity-check has been passed, then the code sequence and its putative nucleotide decoding are valid. If the syndrome has non-zero entries then an error has been detected.

When an error is known to have occurred but is ambiguous, it may be mistakenly ‘corrected’ by applying the wrong error type. The equivalence class contains the possible simple error types that could have caused the observed syndrome but only one of them is the one that actually occurred; applying any to the observed sequence will result in a valid code sequence. We can determine the pattern of nucleotide errors that mistaken correction will result in: if the observed sequence with (unknown) error _*e*1_ is *c* + _*e*1_ and the correction _*e*2_≠_*e*1_ is applied, then converting back to a nucleotide sequence using the inverse of the block generator results in $(c+e_{1}+e_{2})G^{-1}$. The difference between the nucleotide translation of the corrected sequence and the correct nucleotide sequence is $(e_{1}+e_{2})G^{-1}$ — the pattern of error induced. Note that the pattern does not depend on the correct nucleotide sequence, but only on the two changes being considered. By examining all possible combination of error types belonging to an equivalence class, the full set of patterns that may occur can be determined. The pattern of error may be a single base change, or a more complicated multi-base change.

### Practical implementation

The theory described examines the worst case where an error can occur anywhere and there is no additional information about which site it is likely to have affected. Real-life performance of the code depends on additional factors. Firstly, the distribution of where errors occur is not uniform, depending for example on the chemical and physical characteristics of the sequencing process, and may not even be independent between positions; the performance of a code may change depending on the error profile. Secondly, but related, the sequencing platform provides quality information in the form of Phred scores [9] that can be used to help locate the position where an error occurred. Values quantifying the probability that a given call is wrong are known as ‘soft information’, compared to the ‘hard information’ of the observed sequence not being a valid encoding.

A convenient way to deal with these extra complications is to simulate encoded sequence under a realistic model of how errors occur and then decode using dynamic programming, on a ‘trellis’ graph that defines all possible decodings and their relative probabilities, to find the most probable call for each position of the decoded sequence [3]. In practice, we need to consider three classes of differences: those due to variants between sequence under study and the reference it is mapped against, those caused by mutations in the original sequence due to polymerase errors during sample preparation (‘generalised error’) and errors made calling the encoded sequence. The first type of difference is of interest and will affect many reads mapping to a single location. Of the remaining two types of difference, the former occurs before the sequence is encoded, so encoding provides no protection and limits the maximum accuracy of the platform; errors of the latter class may be correctable.

In the absence of empirical data for how the distribution of calls and miscalls changes over probes and rounds, and how errors are correlated between positions, we have assumed that errors occur independently for every element of the code sequence and errors are picked uniformly from the three possibilities. The probability of an error for a particular round and cycle of ligation is taken to be equivalent to the quality score from a read sampled at random from a real set of data, implicitly assuming that the error characteristics for alternative probe sets (which depend on the ligation efficiencies of the different pentamers) will be the same as for the ECC probe set. The simulation scheme is as follows: 

1. Sample a fragment from genome (base-space).

2. Mutate bases of fragment independently with equal probability (generalised error).

3. Encode mutated fragment (convert to code-space).

4. Sample qualities from a set of real data.

5. Mutate the encoded sequence with probabilities defined by quality values.

This scheme outlined has an error model similar to that implicitly assumed when analysing real data since information about both alternative calls and correlation between calls has already been lost during processing into a color-space sequence with a single quality value for each position. The scheme does not simulate the occurrence of insertions and deletions but these are relatively rare compared to substitutions and are most likely to be due to errors introduced during sample preparation rather than calling errors.

## Results and discussion

If a read is error-free then it can be decoded unambiguously and the calls from the two-base-encoding chemistry and ECC codes will agree. When an error occurs in any of the two-base-encoding chemistry rounds, it translates into multiple base miscalls. The structure of the ECC code allows such errors to be detected, recovered from, and, in certain circumstances, corrected. The syndrome equivalence classes are defined by the types of error that can occur and so, by examining them, we can classify the cases where errors can be corrected unambiguously and those where additional information is needed to resolve the ambiguity.

Since the elements of the syndrome consist solely of the summation of the observed and expected parity colors, there is a one-to-one correspondence between them and parity colors. An error in a block can only affect two elements of the syndrome, those corresponding to the two parity colors that overlap the block’s first and last elements. We refer to these as the upstream and downstream syndromes, respectively, so it is sufficient to concentrate on how errors occur in only one block. All statements we make about the error correcting properties of the encoding are on a per-block basis, so a code that can correct one error per block can correct multiple errors if spread between blocks. The final block only has a upstream syndrome and so has more limited error correction.

The value of the syndromes for all possible single-color errors that could occur in a block or its parity colors are shown in Table 2. For example, an error of +1 in the second color produces the syndrome *βα*, identical to that produced by an error of +1 in the third position. If a syndrome is unique (for example, syndrome *ββ* arises only from error + *β* in position _*c*5_) then, assuming only a single error has occurred in that block or parity color, that error can be determined and so corrected. Note that while errors in parity color _*p*−_, overlapping a block and the previous one, appear to have unique syndromes, this parity color is also _*p* + _ for the previous block and so the syndrome can actually be caused by multiple different errors.

**Table 2:** Syndromes for ECC generator

**Error**	**Interpretation**	**Position**										**Equivalence**
**Type**		**_*p* *−*_**	**_*c*1_**	**_*c*2_**	**_*c*3_**	**_*c*4_**	**_*c*5_**	**_*p* * + *_**				**classes**
+ *β*	Complement	*β*0	0*β*	*α*1	*α*1	0*β*	*ββ*	0*β*				{_*c*5_}
+ *α*	Transition	*α*0	0*α*	1*β*	1*β*	0*α*	*αα*	0*α*				{_*c*2_,_*c*3_}
+1	Transcomplement	10	01	*βα*	*βα*	01	11	01				{_*c*1_,_*c*4_,_*p* + _}

All syndromes caused by a single error in a block of five code letters (_*c*1__*c*2__*c*3__*c*4__*c*5_) and two parity letters (_*p*−__*p* + _) of the SOLiD ECC code. Each row in the table corresponds to a specific type of error at the given position of the code word, with the ‘interpretation’ of an error type being the effect it would have when considered as a nucleotide substitution. The table entries are the values of the relevant elements of the syndrome, corresponding to the upstream and downstream parity checks for the block for each error type. The error type +0 is not shown since it represents no error; its syndromes would be 00 at all positions. The equivalence classes are listed separately and do not correspond to specific error types. Note that _*p*−_is also _*p* + _for the preceding block.

The syndrome equivalence classes show the types of single-color error that cannot be disambiguated without additional information; a error has been detected but is not correctable. For example, the syndromes 0*β*, 0*α* and 01 can be generated by single errors at _*c*1_, _*c*4_ or _*p* + _, and so errors at these positions cannot be distinguished. The equivalence classes define the possible simple changes to the observed sequence that will produce a valid sequence that can be decoded into a string of bases, only one of which produces the correct sequence. When an error is wrongly corrected, the code sequence is changed in two places, the original error and the correction; the structure of the code ensures that the changes should be complementary (both ‘+1’ or both ‘ + *α*’, for example).

Using the inverse of the block generator matrix, the pattern of changes that are induced in base-space by miscorrections to the observed sequence can be derived. If errors occur and are miscorrected, such that the difference between the correct and observed values of the data colors is *d*, then the pattern of differences *p* induced in base-space is given by $p=dG_{\text{Block}}^{-1}$. For example, an error of type + *α*at the second position might be erroneously corrected by an error of type + *α* at the third position, so the differences are 0*αα*00 and the pattern induced in base-space is 00*α*00 (the sum of the second and third rows of $G_{\text{Block}}^{-1}$ multiplied by *α*). If this occurred to the nucleotide sequence ATGCG then the sequence ATACG would result.

All patterns induced by single-color errors are listed in Table 3; for example, an error of type + *α* at the first position may be wrongly corrected at either the fourth position or the parity color, leading to the triple error 0*ααα*0 or the quadruple error 0*αααα*, respectively. Note that no pattern ever affects the first position of the block; the structure of the matrix $G_{\text{Block}}^{-1}$ shows that this position can only be changed by errors in the second, third or fifth positions: single-color errors at the fifth position can be always be unambiguously corrected, and errors at the second or third positions result in either corrections or changes that cancel at the first position.

**Table 3:** Patterns of error for the ECC generator

**Positions**	**Change**	**Pattern**
_*c*2_,_*c*3_	Single	00100
_*c*4_,_*p* + _	Single	00001
_*c*1_,_*c*4_	Triple	01110
_*c*1_,_*p* + _	Quadruple	01111

All possible patterns of error caused in corrected base-space sequence by a single wrongly corrected error in the observed sequence of type +*d* (where *d*=1, *α*or *β*). The ‘Positions’ column indicates all possible pairs of positions at which an error can occur and be wrongfully corrected; it is not necessary to identify which member of the pair corresponds to which form of error as the resulting pattern is the same. In all cases the error pattern should be multiplied element-wise by the error type (*d*) to get the actual pattern (e.g. a wrongly corrected + *β*error at _*c*2_results in an error pattern of 00*β*00).

One notable feature of these more complex errors is that the error type is the same at all affected positions, a property that might help distinguish sequencing errors from genuine variants if the reads are later mapped to a reference. By adding the mapped read to the reference in GF_4_, the pattern should be evident if it was due to simple miscall. If the pattern is not evident, the differences are either due to sequence variants or multiple miscalls.

While the consideration of syndromes provides useful information about a code’s properties and syndrome decoding is computationally efficient, it is not a replacement for probabilistic methods of decoding since the latter incorporates the quality information about each call and can use it to make better decisions about correction. However, syndrome decoding techniques may still be of use in conjunction with the more computationally expensive probabilistic methods since the syndrome provides a quick test of whether the observed sequence is valid (no correction needed). Syndrome equivalence classes could also be used to restrict the paths through a trellis to a plausible set, providing a heuristic to speed up the dynamic programming algorithm to find the most probable decoding.

### Theoretical bounds

We have shown the type of errors that the ECC code can correct but have not yet addressed whether it is optimal. Before examining specific alternative codes, it is interesting to look at what can possibly be achieved and there are several mathematical results that restrict the performance of any code. Firstly the Hamming [10], Johnson [11] and Singleton [12] bounds place an upper limit on the number of errors that a code can hope to detect or correct but codes meeting these bounds may not exist; the Gilbert–Varshamov bound [13,14] is a lower limit on the performance of the best code that does exist.

For reads of 50 bases encoded into 60 letters (as with the ECC code), the lower bound guarantees that a code exists that can detect any two errors and correct any single error. The upper bounds show that no code can guarantee to correct more than three errors. For reads of length 75 bases encoded into 90 letters, then a code exists that can detect and correct any two errors but no code can guarantee to detect and correct more than four errors. There is no guarantee that a convolutional code can meet this bound and the two most common classes of codes that come close to attaining these bounds, Turbo codes [15] and Low-Density Parity-Check codes [16], require long-range dependencies between positions in the sequence and so cannot be implemented in any plausible sequencing chemistry.

### Alternative chemistries

Examining the syndromes in Table 2, we notice that, despite many possible single errors having ambiguous syndromes, not all syndromes are present: the three syndromes *αβ*, *β*1 and 1*α* do not occur. The missing syndromes are not truly unused, being generated by multiple errors, but the failure to use them to distinguish single errors suggests that there may exist alternative codes with a greater ability to correct single calling errors. The advantage would derive from using the extra syndromes to partition single-color errors more evenly into equivalence classes. Such alternative codes can be analysed using the same techniques as the ECC code.

Rather than consider all possible convolutional codes, we will focus our attention on a subset that satisfy some reasonable restrictions inspired by the reality of the SOLiD platform. It is desirable that any new chemistry would be backwards compatible with the two-base-encoding chemistry, meaning that the first five rounds of sequencing must use an unaltered two-base-encoding probe set. This backwards compatibility restriction is equivalent to requiring that a new code must have an unpunctured color stream.

While the number of rounds and probe sets could be varied, and probes of differing lengths could be used, to remain comparable to the current ECC chemistry we will focus only on chemistries where a single additional round (and so only one additional probe set) will be used and the probes remain based on pentamers. Analogous to the code structure shown in figure 1, alternative codes that can be implemented with a single additional round are punctured so only every fifth element of the second stream is produced.

By redefining the boundaries of the block structure, the number of different alternative codes that need to be considered can be further reduced: a code whose probe generator starts with one or more 0s is identical to one starting at the first non-zero element with the tail padded with zeros. The probe generator of any code that starts with *α* or *β* can be written as *αW* or *βW*, where *W * is the probe generator of a code starting with 1, and the linearity of convolutional codes ensures that the two codes have identical sets of code words: although the mapping between nucleotide sequence and encoded sequence will differ, the error correcting characteristics will be the same.

The block generator for alternative codes that satisfies all the restrictions, and its inverse, can be written as

(4) $$G_{\mathrm{alt}}=\left(\begin{array}{c} 1\;0\;0\;0p_{1}\\ 1\;1\;0\;0p_{2}\\ 0\;1\;1\;0p_{3}\\ 0\;0\;1\;1p_{4}\\ 0\;0\;0\;1p_{5} \end{array}\right)G_{\mathrm{alt}}^{-1}=\left(\begin{array}{c} 1\;0\;0\;0x_{1}y^{-1}\\ 0\;1\;0\;0x_{2}y^{-1}\\ 0\;0\;1\;0x_{3}y^{-1}\\ 0\;0\;0\;1x_{4}y^{-1}\\ 0\;0\;0\;0y^{-1} \end{array}\right)L$$

where _*p*1__*p*2__*p*3__*p*4__*p*5_ is the generator for the second set of probes, *L* is the matrix whose upper triangle consists of 0 and whose diagonal and lower triangular elements are all 1, *x*=*Lp* and *y*=_*x*5_. The ECC code has the probe generator _*p*1__*p*2__*p*3__*p*4__*p*5_=1*β*0*β*0. The inverse generator only exists when *y* is invertible (i.e. *y*≠0); when *y* is not invertible, blocks of colors cannot be individually inverted and the syndrome analysis is not applicable. Due to the structure of *L*, requiring *y* to be invertible is the same as the sum of the elements of the probe generator not being zero.

One further restriction will be placed on the probe generator of alternative codes: the ECC probe generator contains 0 at its fifth position and this is probably a consequence how accurately pentamers with differing final bases can be ligated to the sequence. Codes whose generators use the final position might theoretically have better error correction properties but the increased rate of calling errors, due to incorrect probes being ligated, may cancel any improvement their use may offer; consequently, we will require _*p*5_=0.

There are 48 probe generators satisfying all the restrictions and the invertibility condition, of which the syndromes and equivalence class for two interesting alternatives are shown in Table 4. The first code has generator _*p*1__*p*2__*p*3__*p*4__*p*5_=10*β*00 and has similar equivalence classes to the ECC code but only uses the first three positions of the generator. While the set of syndromes for single-color errors is also incomplete (syndromes 1*β*, *α*1 and *βα* are unused) and thus the error correcting properties will be similar to the ECC code, the shorter length of the generator means that calculations on the trellis, to determine the most probable decoding, can be carried out four times quicker.

**Table 4:** Syndromes for alternative codes

**Generator**	**Error**	**Position**										**Equivalence**
	**type**	**_*p* *−*_**	**_*c*1_**	**_*c*2_**	**_*c*3_**	**_*c*4_**	**_*c*5_**	**_*p* * + *_**				classes
10*β*00	+ *β*	*β*0	1*α*	1*α*	0*β*	0*β*	*αα*	0*β*				{_*c*5_}
	+ *α*	*α*0	*β*1	*β*1	0*α*	0*α*	11	0*α*				{_*c*1_,_*c*2_}
	+1	10	*αβ*	*αβ*	01	01	*ββ*	01				{_*c*3_,_*c*4_,_*p* + _}
1*β*010	+ *β*	*β*0	*α*1	1*α*	1*α*	0*β*	11	0*β*				{_*c*1_},{_*c*5_}
	+ *α*	*α*0	1*β*	*β*1	*β*1	0*α*	*ββ*	0*α*				{_*c*2_,_*c*3_}
	+1	10	*βα*	*αβ*	*αβ*	01	*αα*	01				{_*c*4_,_*p* + _}

All syndromes caused by a single error in a block of five code letters (_*c*1__*c*2__*c*3__*c*4__*c*5_) and two parity letters (_*p*−__*p* + _) for codes with the specified generator. Each row corresponds to a specific type of error at the given position of the code word and the table entries are the values of the relevant elements of the syndrome corresponding to the upstream and downstream parity checks for the block for each error type. The error type +0 is not shown since it represents no error. The equivalence classes are listed separately and do not correspond to specific error types. Note that _*p*−_is also _*p* + _for the preceding block.

The second alternative code shown in Table 4, with probe generator 1*β*010, uses all possible syndromes and potentially has better error correcting properties than the ECC code. Comparing the equivalence classes of the new code to those for the ECC code, the new code uses the extra syndromes to split the largest class into two. Whereas the ECC code is unable to distinguish errors at the first, fourth or parity positions, the new code can unambiguously correct errors at the first position and the ambiguity of fourth and parity position errors is reduced.

Table 5 shows the error patterns induced by single-color errors for the two alternative codes considered. Note the reduced number of base-space errors relative to the ECC code (Table 3).

**Table 5:** Patterns of error for alternative codes

**Generator**	**Positions**	**Change**	**Pattern**
10*β*00	_*c*1_,_*c*2_	Single	01000
	_*c*3_,_*c*4_	Single	00010
	_*c*3_,_*p* + _	Double	00011
	_*c*4_,_*p* + _	Single	00001
			
1*β*010	_*c*2_,_*c*3_	Single	00100
	_*c*4_,_*p* + _	Single	00001

All possible patterns of error caused in corrected base-space sequence by a single, wrongly corrected error in the observed sequence for code with given generator. An error occurs at one of the positions in the ‘Positions’ column; the other member of ‘Positions’ is where the observed sequence is wrongfully corrected. As in Table 3, all error patterns should be multiplied element-wise by the error type (1, *α*or *β*) to get the actual pattern.

### Simulations

The theory described suggests that the code with probe generator 1*β*010 is more powerful than the ECC code but this does not necessarily mean that it performs better in practice since actual performance will depend on the distribution of different types of error and at which positions they occur. To help quantify the difference in performance between codes, they were compared on artificial data, simulated so that it had an error profile similar to real data but for which the original sequence of bases is known, the error profile being estimated by mapping to a SNP-corrected genome.

One million fragments were sampled uniformly from the positive strand of the genome of *E. coli* DH10b with qualities being sampled from a real sequencing run of the same genome. The probability of generalised error was set to be equivalent to _*Q*34_, close to observed values for prepared samples. Three sets of reads were produced, using different codes on the same set of fragments to generate the ECC information, and sequence was called into both base-space and color-space using the Maximum A Posteriori (MAP) criterion. Reads were then mapped to an appropriately encoded reference using the BWA aligner [17] with an edit distance of five. Results, in terms of the total proportion of reads mapping and the proportion mapping with a given number of errors, are shown in Table 6.

**Table 6:** Number of errors for simulated data

**Space**	**Probe**	**Percentage**	**Percentage mapped with 0 – 5 errors**					
	**generator**	**mapped**	**0**	**1**	**2**	**3**	**4**	**5**
Color	None	77.1	38.4	16.7	10.5	6.2	3.6	1.8
	1*β*0*β*0	78.4	48.6	10.2	9.7	5.0	3.2	1.7
	10*β*00	78.3	48.4	10.3	9.7	5.0	3.2	1.7
	1*β*010	78.3	49.8	9.1	9.7	4.9	3.2	1.7
Base	None	47.2	38.4	2.3	1.6	1.8	1.8	1.2
	1*β*0*β*0	64.3	49.6	4.7	2.1	3.1	2.6	2.1
	10*β*00	65.5	49.4	6.2	3.0	2.3	2.4	2.2
	1*β*010	64.9	50.8	5.0	2.2	2.4	2.4	2.2

Percentage of reads mapped (five edits or fewer), and mapped with a given number of errors, for one million simulated reads using the codes with probe generators as specified. For comparison, figures are also given using only the two-base-encoding probe set (probe generator ‘None’). Since color-space reads have their first position trimmed before mapping to produce reads 49 colors long, the percentages of mapped reads and reads with a given number of errors are slightly inflated compared to those given for base-space reads.

Using the ECC information (probe generator 1*β*0*β*0) to help call reads makes a considerable improvement in both color-space and base-space. Without any correction, 77.1% of simulated reads map to the reference in color-space and only 38.4% of these are perfect, with a further 16.7% having one error. After correction, 48.6% of reads are perfect, with 10.2% containing one error. In base-space, the number of mapped reads increases from 47.2% to 64.3%. This large increase is due to correcting single-color errors that would otherwise induce multiple base errors and prevent the read from being mapped using the ‘five differences or fewer’ criterion.

All three codes perform better than uncorrected sequence in both color-space and base-space. There was little difference between their performance in absolute terms, although the code generated by 1*β*010 produced more error-free reads than the other two. This small absolute difference disguises a larger increase in the proportion of corrected reads: under the 1*β*0*β*0 code, 10.2% of reads were corrected to being perfect compared to 11.4% for the 1*β*010 code, an 11.8% relative improvement even though the absolute improvement is only 1.2%. The 10*β*00 code produces more mappable base-space sequence than either of the other codes and the reason for this may be in the pattern of errors in base-space that single-color errors make: comparing the patterns in Tables 3 and 5 shows that, for the two alternative codes, single-color errors predominantly cause a single base error when wrongfully corrected, rather than more complex errors, and so there are fewer base-space errors in total.

While the code generated by 1*β*010 does perform better than the ECC code, the improvement is not especially dramatic despite the syndrome equivalence classes suggesting superior error-correcting properties. The decoding algorithm uses soft information as well as the hard information from color calls when determining the most probable base at each position of decoded sequence and this is a possible explanation for the small difference between the performance of the codes. The equivalence classes narrow down the possible errors but, rather than randomly picking the correction, MAP decoding uses the quality information to guide the choice and the right correction might be picked the majority of the time even without this extra assistance.

## Conclusions

The addition of the Exact Call Chemistry to the SOLiD platform enables many sequencing errors to be detected that would otherwise would pass unnoticed; this in itself provides useful information about the accuracy of reads. Without using quality information, the ability of the Exact Call Chemistry to correct sequencing errors is limited but the number of possible options can often be drastically reduced. The quality of the calls for the encoded sequence can then be used to choose between the options for correction, leading to genuine ability to correct sequencing mistakes. The five-base length of the block places a limit on how frequently errors can occur before the encoding can no longer offer protection. In our simulations, the number of perfect color-space reads increases by 27% when error correction is performed, the majority of these corrections being single errors that might otherwise degrade the base-space translation of the read. The error correction capability offered by the ECC results in measurable gains for the SOLiD platform but there is a trade-off between the extra time and expense and the improvements possible through increasing coverage using additional sequencing runs.

The complex triple and quadruple errors that can be induced in the final nucleotide reads by miscorrection of the observed sequence are not well represented by the Phred error model [9], where each site is assigned an individual quality independent of other sites. This may have consequences for downstream analyses that assume the Phred model is a good representation of the probability that a particular site is in error. The patterns of error induced by the largest equivalence class of the ECC code may allow some sequencing errors to be distinguished from genuine variants after mapping to a nucleotide reference. The power of this approach is unlikely to be good and so it will not be an adequate replacement for mapping and variant calling using a more appropriately encoded reference. One possibility would be to encode the reference using the ECC code and match directly against that, using all the available information but making variant calling difficult due to the complex structure of the data. A simpler alternative would be to decode into two-base-encoding colors, using the extra ECC calls to correct the color calls. The corrected color reads can then be mapped against a color-encoded reference using the many tools already developed.

The variation in the ability of the ECC to correct errors at different positions in each block suggests a simple improvement to the platform that would enable it to recover from problematic rounds. Consider, for example, that one of the initial five rounds has experienced some form of gross failure, perhaps due to a bad wash or incomplete melting of the previous primer and sequence from the template, so that every cycle in that round is of poor quality. The priming of the final round could be adjusted to start at a different position of each block, chosen to maximise the chance of correcting previous errors. For the ECC code, the priming would be chosen to ensure that calls from the bad round coincide with positions two or three of the final round (the equivalence class with two elements); the alternative code generated by 1*β*010 would be primed so that the errors coincide with the first position and correction could be guaranteed. This technique could also help recover from transient errors, like bubbles in the buffer preventing calls being made for a large number of clusters on a particular cycle.

The alternative codes that we describe have equal or superior error correction ability to that of the ECC but the creation of a new probe set is a considerable investment and a more thorough search of alternatives codes should be undertaken before the expense is incurred. All the alternative codes considered satisfy a number of restrictions, several of which could be relaxed to improve the error correction properties. Firstly, more rounds could be used, slowing the sequencing process down but providing more redundancy with which to correct errors. Secondly, the architecture of the encoder was constrained to be backwards compatible with the two-base-encoding sequencing chemistry, completely defining one unpunctured stream. A completely new chemistry could vary both probe sets and also change the puncturing matrix, allowing much more flexibility over the design of the code architecture and potentially creating codes that are capable of unambiguously correcting multiple errors.

While many sequencing errors can be corrected, their occurrence is non-uniform, being more frequent in the later cycles of each round. This leads to a tendency for the poorest reads to contain multiple errors in close proximity. Such bursts of errors cannot be successfully corrected, so the advantage of the ECC is limited to generally high quality reads containing a few sparsely distributed errors. Perhaps the major advantage of the new chemistry is that the sequencing platform can produce reliable nucleotide sequence, without the possibility of a single error causing a catastrophic decoding failure, which allows the reads produced to be analysed with the wealth of tools available that assume nucleotide sequence.

### Software

Software reimplementing the decoding algorithm for the two-base- and four-base- encodings (SOLiD ECC), as well as the alternative encodings described, on a trellis to find both the maximum likelihood (Viterbi decoding) and maximum a posteriori [3] (forwards/backwards decoding) nucleotide sequence is available at http://www.ebi.ac.uk/goldman-srv/solid/, distributed under version 3 of the GNU General Public Licence, as is software to simulate two- and four-base encoded reads and some utility functions to manipulate convolutional codes in GF_4_. This software is provided solely for the purposes of reproducibility.

## Competing interests

This work was supported in part by Wellcome Trust Technology Development grant WT088151MA and a grant to the EBI from Life Technologies Corporation. Life Technologies Corporation had no input into the development of the study, article preparation or decision to publish. No other competing interests are declared.

## Authors contributions

The study was conceived and planned by both authors. The analysis of the Exact Call Chemistry coding was done by TM, who also drafted the manuscript. Both authors read, edited and approved the final manuscript.
